# Rheumatoid arthritis and the risk of postpartum psychiatric disorders: a Nordic population-based cohort study

**DOI:** 10.1186/s12916-023-02837-3

**Published:** 2023-04-03

**Authors:** Min Luan, Fen Yang, Maohua Miao, Wei Yuan, Mika Gissler, Elizabeth V. Arkema, Donghao Lu, Jiong Li, Krisztina D. László

**Affiliations:** 1 NHC Key Laboratory of Reproduction Regulation (Shanghai Institute for Biomedical and Pharmaceutical Technologies), Fudan University, Shanghai, China; 2 Department of Global Public Health, Karolinska Institutet, Tomtebodavägen 18A, 171 77 Stockholm, Sweden; 3 Clinical Research Center, Shanghai Sixth People’s Hospital Affiliated to Shanghai Jiao Tong University School of Medicine, Shanghai, China; 4 Department of Knowledge Brokers, Finnish Institute for Health and Welfare, Helsinki, Finland; 5 Academic Primary Health Care Centre, Region Stockholm, Stockholm, Sweden; 6 Department of Molecular Medicine and Surgery, Karolinska Institutet, Stockholm, Sweden; 7 Clinical Epidemiology Division, Department of Medicine Solna, Karolinska Institutet, Stockholm, Sweden; 8 Unit of Integrative Epidemiology, Institute of Environmental Medicine, Karolinska Institutet, Stockholm, Sweden; 9 Department of Clinical Medicine-Department of Clinical Epidemiology, Aarhus University, Aarhus, Denmark; 10 Department of Public Health and Caring Sciences, Uppsala University, Uppsala, Sweden

**Keywords:** Postpartum psychiatric disorders, Postpartum depression, Rheumatoid arthritis, Cohort study

## Abstract

**Background:**

Postpartum psychiatric disorders (PPD) are common complications of childbirth. A common explanation for their development is that the psychological, hormonal, and immune changes associated with pregnancy and parturition may trigger psychiatric symptoms postpartum. Rheumatoid arthritis (RA) is characterized by abnormalities in the activity of the hypothalamic–pituitary–adrenal axis and of the immune system, but its association with PPD is unknown. We analyzed whether women with RA before childbirth have an increased risk of PPD.

**Methods:**

We conducted a large population-based cohort study including mothers of singleton births in the Danish (1995–2015), Finnish (1997–2013), and Swedish Medical Birth Registers (2001–2013) (*N* = 3,516,849). We linked data from the Medical Birth Registers with data from several national socioeconomic and health registers. Exposure was defined as having a diagnosis of RA before childbirth, while the main outcome was a clinical diagnosis of psychiatric disorders 90 days postpartum. We analyzed the association between RA and PPD using Cox proportional hazard models, stratified by a personal history of psychiatric disorders.

**Results:**

Among women without a history of psychiatric disorders, the PPD incidence rate was 32.2 in the exposed and 19.5 per 1000 person-years in the unexposed group; women with RA had a higher risk of overall PPD than their unexposed counterparts [adjusted hazard ratio (HR) = 1.52, 95% confidence intervals (CI) 1.17 to 1.98]. Similar associations were also observed for postpartum depression (HR = 1.65, 95% CI 1.09 to 2.48) and other PPD (HR = 1.59, 95% CI 1.13 to 2.24). Among women with a history of psychiatric disorders, the incidence rate of overall PPD was 339.6 in the exposed and 346.6 per 1000 person-years in the unexposed group; RA was not associated with PPD. We observed similar associations between preclinical RA (RA diagnosed after childbirth) and PPD to those corresponding to clinical RA.

**Conclusions:**

Rheumatoid arthritis was associated with an increased PPD risk in women without, but not in those with a psychiatric history. If our findings are confirmed in future studies, new mothers with RA may benefit from increased surveillance for new-onset psychiatric disorders postpartum.

**Supplementary Information:**

The online version contains supplementary material available at 10.1186/s12916-023-02837-3.

## Background

Postpartum psychiatric disorders (PPD), with an overall prevalence of 11–15%, are among the most common complications that women experience in relation to childbirth [1]. They may disrupt the developing attachment between the mother and the child and may have important social and health-related consequences for affected women and their families (e.g., increased risks of subsequent chronic medical conditions, premature death, self-harm or suicide, and infanticide) [2, 3]. Postpartum depression is one of the most common PPDs with an estimated prevalence of 5–15% [4, 5]. A number of risk factors for PPD have been identified, including genetic factors, history of psychiatric disorders, primiparity, and pregnancy complications [6, 7]. However, the etiology of PPD is considered very complex and to a large extent unknown.

Immunological mechanisms have been implicated in the pathogenesis of psychiatric disorders both in general population and in postpartum women [8, 9]. One of the immune-related disorders, rheumatoid arthritis (RA), has a prevalence of 1–2% in the general population and affects mostly women aged 30 or older [10]. A large number of studies based on general population samples have documented an increased prevalence of psychiatric disorders in patients with RA [8, 11]. RA is favorably influenced by pregnancy due to the suppression of autoimmunity during pregnancy [12]. After delivery, the relatively immunologically suppressed state of pregnancy shows a rebound and there is a shift from an anti- to a pro-inflammatory state [12]. This may contribute to the exacerbation of the activity of RA. Moreover, women without adequately suppressed immune function and/or prolonged and exaggerated inflammatory response postpartum may be particularly sensitive to difficulties in emotional regulation during the postpartum period [9]. Therefore, a link between RA and new-onset PPD or postpartum relapse in patients with a history of psychiatric disorders is plausible. Furthermore, women with RA before childbirth have an increased risk of pregnancy complications and adverse birth outcomes [13], which may also increase PPD risk. A large cohort study has also shown that inflammatory bowel disease, an immune-mediated inflammatory disease similar to RA, is associated with an increased risk of new-onset PPD [5]. In contrast, another study reported a lower risk of new-onset depression in the year after birth in women with juvenile idiopathic arthritis than in their unexposed counterparts [14]. To our knowledge, no previous study investigated the association between RA before childbirth and the risk of PPD.

In this large cohort study including women from three Nordic countries, we investigated whether women with RA before childbirth had an increased risk of PPD. Since women with previous psychiatric disorders have an increased risk of a relapse postpartum and a more heterogeneous PPD phenotype than women without such a history [15], we also analyzed whether the association differs according to a history of psychiatric disorders.

## Methods

### Study population and design

This population-based cohort study used data from several nationwide registers in Denmark, Finland, and Sweden, where every resident has a unique personal identification number, allowing linkage between nationwide registers [16]. The registers used for this study are described in detail in Additional file 1: Table S1. Our study population consisted of women with singleton births in the nationwide Medical Birth Registers in Denmark during 1995–2015 [17], in Finland during 1997–2013 [18], and in Sweden during 2001–2013 [19]. We first identified all births in the Medical Birth Registers for the Danish and Swedish cohorts, while for the Finnish cohort, due to the data protection rules of the data holder authority, we could include a randomly selected 90% of the births from the register. The study periods were chosen based on considerations regarding the years when information on both inpatient care and specialized outpatient care was available in the country’s patient register. Our study population consisted of mothers of the abovementioned 3,516,849 singleton births.

### Measures

#### Rheumatoid arthritis

Information on the diagnoses of RA was obtained from the Danish Hospital Register during 1977–2016 [20], the Finnish Hospital Discharge Register during 1987–2015 [21], the Swedish Patient Register during 1968–2014 [22], and the Swedish Medical Birth Register during 2001–2013 [19]. The diagnosis of RA was identified by International Statistical Classification of Diseases and Related Health Problems (ICD) codes (Additional file 1: Table S2). We classified women as exposed to RA if they were diagnosed with either a primary or a secondary diagnosis of RA prior to giving birth. Women without a record of RA before childbirth were considered unexposed. The date of the first hospitalization or the first visit to the outpatient clinic associated with the RA diagnosis was considered the date of the clinical diagnosis of RA.

Prior to the development of RA, there is a period with an increase in levels of disease-related biomarkers similar to that of clinical RA, including autoimmune antibodies and a wide range of inflammatory cytokines and chemokines (termed as “preclinical RA”) [23]. Thus, we also defined exposure to clinical RA (i.e., RA diagnosed before childbirth) and preclinical RA (i.e., RA first diagnosed after childbirth) using the date of first RA diagnosis to disentangle their effects on PPD [24].

#### Postpartum psychiatric disorders and a history of psychiatric disorders

Information on psychiatric diagnoses was retrieved from the Danish Hospital Register, the Danish Psychiatric Central Research Register [25], the Finnish Hospital Discharge Register, and the Swedish Patient Registers (Additional file 1: Table S1). Our primary outcome was a first diagnosis of a psychiatric disorder (ICD 10: F00-F99) given as primary or secondary diagnosis in hospital inpatient or outpatient care in the first 90 days after delivery. We chose this period as it is the most vulnerable time after birth [26]. To address concerns about statistical power and to consider that there was likely a lag between onset of symptoms and timing of diagnoses, we conducted analyses with a more widely defined postpartum period, i.e., the first 365 days postpartum as our secondary outcome. Each woman was followed from the date of delivery until a PPD, death, emigration, or 90/365 days postpartum, whichever came first. We further categorized PPDs as (1) postpartum depression (given its clinical relevance and prevalence) and (2) other PPD.

We defined a personal history of psychiatric disorders as having at least one record of psychiatric disorders in inpatient or outpatient care prior to delivery; the corresponding ICD codes used are shown in Additional file 1: Table S2. A family history of psychiatric disorders was also identified using the same strategy (i.e., based on biological parents identified in the Danish Civil Registration System and the Swedish Multi-Generation Register).

#### Other covariates

From the Medical Birth Registers, we obtained information on maternal age at delivery, maternal body mass index (BMI) in early pregnancy (available in Sweden during the whole study period and in Denmark since 2004), calendar year of childbirth, gestational age, birth weight, parity, and stillbirth. Small for gestational age (SGA) was defined as being below the 10th percentile of the birth weight for gestational age distribution based on the Swedish sex-specific reference curve for normal fetal growth [27]. We identified maternal highest education at the time of delivery from the Danish Integrated Database for Longitudinal Labor Market Research and the Education Register in Sweden and Finland, and maternal marital status at delivery from the Danish Civil Registry System, the Swedish Total Population Register, and the Finnish Medical Birth Register. The Danish Integrated Database for Longitudinal Labor Market Research and the Swedish Medical Birth Register provided data on the mothers’ country of origin.

Information on maternal diabetes before or during pregnancy, pre-eclampsia, and placental abruption during the index pregnancy was obtained from the Danish Hospital Register, the Finnish Hospital Discharge Register, and the Swedish Medical Birth Register. A full list of ICD codes used to define the above medical conditions is presented in Additional file 1: Table S2. Information on psychiatric medications was obtained from the Swedish Prescribed Drug Register (since 2005) and the Finnish Prescription Register (since 1995) using the Anatomical Therapeutic Classification codes N05 and N06A [28, 29].

### Statistical analyses

We performed Cox proportional hazard models to estimate crude and adjusted hazard ratios (HRs) with 95% confidence intervals (CIs) for the associations between maternal RA and the risk of PPD. Visual inspection of the log–log curves suggested no evidence of violation of the assumption of proportional hazards. Since we found evidence that a history of psychiatric disorders modified the association between RA and PPD, we also presented results stratified by personal history of psychiatric disorders before childbirth. In the main multivariable model, we adjusted for a priori selected confounders which were available in all three countries, i.e., factors associated with both RA and PPD, but not on the causal pathway between RA and PPD: maternal age at delivery (< 25, 25–29, 30–34, or ≥ 35 years), calendar year of childbirth (in 5-year intervals during 1995–2015), parity (primiparous or multiparous), maternal highest education (primary and lower secondary, upper secondary, or university), and marital status at delivery (married/registered partnership vs. not). Since information on maternal BMI in early pregnancy (< 18.5 kg/m^2^ for underweight, 18.5–24.9 kg/m^2^ for normal weight, 25.0–29.9 kg/m^2^ for overweight, and ≥ 30.0 kg/m^2^ for obesity), family history of psychiatric disorders (yes or no), and country of origin (Denmark/Sweden or other countries) was not available for all study participants, we adjusted for these characteristics in separate models among those with available data on these variables.

We performed separate analyses for exposure to clinical RA and to preclinical RA; these analyses aimed to discriminate the effect of the potential treatment and care from the effect of RA alone because we assumed that women with preclinical RA did not receive specific RA treatment or care before childbirth.

We performed a number of sensitivity analyses. To control for confounding from shared familial factors, we performed an analysis with a sibling design involving women and their sisters from the Danish and Swedish Medical Birth Registers. The sister pairs were identified based on the same ID of their biological mother and were included if both had at least one singleton delivery during the study period. We ran stratified Cox proportional hazards models in a subsample of 666,794 maternal-sister pairs, with a separate stratum for each maternal-sister pair. Only maternal-sister pairs discordant for RA and PPD were informative and contributed to the risk estimates. We additionally adjusted one by one for pre-eclampsia, placental abruption, SGA [27], preterm birth (gestational age < 37 weeks), and stillbirth to investigate whether pregnancy complications and adverse delivery outcomes contributed to the association of interest. Besides, to address potential biases related to selective fertility [30], i.e., that women with a previous PPD or other pregnancy complications or adverse birth outcomes would refrain from engaging in a new pregnancy, we performed analyses restricted to primiparous women. Since diabetes and pre-eclampsia may be regarded as immune-related diseases and were associated with increased risks of PPD [31, 32], we performed stratified analyses and tests of interaction between RA and preeclampsia, and tests of interaction between RA and diabetes to examine the potential effect modification by these conditions. To consider differences over time in the care of pregnant women with RA or in the prevalence of PPD (Additional file 1: Fig. S1), we also conducted a propensity score-matched (PSM) analysis to further balance the exposure groups on potential confounders. The propensity scores were estimated using logistic regression as the probability of exposure to RA given baseline characteristics such as calendar year of childbirth, parity, maternal age at delivery, and study country. Each woman exposed to RA was matched to an unexposed woman on the propensity scores by using greedy match algorithms with a ratio of 1:1. We then repeated the analyses in the PSM sub-cohort with additional adjustments for the woman’s highest education and marital status at delivery. To increase the specificity of the definition of preclinical RA, we also performed analyses with a narrowly defined preclinical RA, i.e., RA diagnoses in the year after delivery. To account for the effect of use of psychiatric medication among women with a history of psychiatric disorders, we analyzed in this sub-cohort the association between RA and PPD after stratifying by a history of psychiatric medication use. Finally, we repeated our main analyses with our secondary outcome, i.e., PPD in the 365 days after delivery.

Statistical analyses were performed using SAS version 9.2. A two-tailed *p*-value of < 0.05 was considered statistically significant.

## Results

Of the 3,516,849 childbirths of women included in our study, 293,371 (8.34%) had and 3,223,478 (91.66%) did not have a history of psychiatric disorders. The incidence rates of PPD in the first 90 days postpartum for the two groups were 346.5/1000 person-years and 19.5/1000 person-years, respectively (Additional file 1: Table S3). Altogether, 8407 (0.24%) births were exposed to maternal RA and 3,508,442 (99.76%) were unexposed (Table 1). Compared to women without RA, women with RA were more likely to be older and better educated and to have higher BMI in early pregnancy and less likely to be married or in a registered partnership at the time of delivery. They were also more likely to have a personal and a family history of psychiatric disorders, as well as pre-eclampsia and placental abruption during the index birth. In addition, exposed mothers were more likely to have preterm births, SGA births, and stillbirths compared to those in the unexposed group (Table 1). Characteristics of women exposed to preclinical RA relative to that of women with clinical RA or unexposed women are shown in Additional file 1: Table S4.

**Table 1:** Characteristics of the study population according to maternal rheumatoid arthritis before childbirth

	Births of women			
	With RA (*N* = 8407, 0.24%)		Without RA (*N* = 3,508,442, 99.76%)	
	No	%	No	%
**Maternal characteristics**				
Study country				
Denmark	4297	51.11	1,291,985	36.83
Finland	215	2.56	912,370	26.00
Sweden	3895	46.33	1,304,087	37.17
Maternal age at delivery (years)				
< 25	735	8.74	539,085	15.37
25–29	2271	27.02	1,116,203	31.81
30–34	3163	37.62	1,188,931	33.89
≥ 35	2238	26.62	664,223	18.93
Maternal country of origin ^a^				
Denmark/Sweden	4262	52.03	1,494,124	57.55
Other country	3929	47.96	1,099,794	42.37
Unknown	< 5	0.01	2154	0.08
Maternal BMI in early pregnancy (kg/m^2^)^a^				
< 18.5	204	2.49	57,841	2.23
18.5–24.9	3728	45.51	1,084,402	41.76
25.0–29.9	1654	20.19	475,472	18.32
≥ 30.0	876	10.69	235,133	9.06
Unknown	1730	21.12	743,224	28.63
Marital status at delivery				
Single, widowed or divorced	4710	56.03	1,761,697	50.21
Married or in registered partnership	3690	43.89	1,712,614	48.82
Unknown	7	0.08	34,131	0.97
Maternal highest education at delivery				
Primary and lower secondary	1226	14.58	792,049	22.58
Upper secondary	3449	41.03	1,235,290	35.21
University	3668	43.63	1,334,893	38.05
Unknown	64	0.76	146,210	4.16
Maternal smoking in early pregnancy				
No	7122	84.72	2,954,987	84.23
Yes	957	11.38	421,187	12.00
Unknown	328	3.90	132,268	3.77
Parity				
Primiparous	3533	42.02	1,441,227	41.08
Multiparous	4874	57.98	2,066,8347 (58. 90)	58. 90
Unknown	0	0.00	868	0.02
Psychiatric disorders before childbirth				
No	7221	85.89	3,216,257	91.67
Yes	1186	14.11	292,185	8.33
Family history of psychiatric disorders^a^				
No	6631	80.94	1,960,464	75.52
Yes	1040	12.70	240,350	9.26
Unknown ^b^	521	6.36	395,258	15.24
Diabetes				
No	7984	94.97	3,349,807	95.48
Yes	423	5.03	158,735	4.52
Pre-eclampsia				
No	7996	95.11	3,410,056	97.20
Yes	411	4.89	98,386	2.80
Placental abruption				
No	8363	99.48	3,496,287	99.65
Yes	44	0.52	12,155	0.35
**Characteristics of the birth**				
Calendar year of birth				
1995–2000	759	9.03	566,190	16.14
2001–2005	2077	24.70	1,048,250	29.88
2006–2010	3017	35.89	1,119,437	31.90
2011–2015	2554	30.38	774,565	22.08
Small for gestational age				
No	7280	86.60	3,115,135	88.79
Yes	1009	12.00	346,001	9.86
Unknown	118	1.40	47,036	1.35
Preterm birth (gestational < 37 weeks)				
No	7675	91.30	3,307,208	94.26
Yes	667	7.93	170,470	4.86
Unknown ^c^	65	0.77	30,764	0.88
Stillbirth				
No	8367	99.52	3,499,154	99.74
Yes	40	0.48	9288	0.26

*RA* rheumatoid arthritis, *No.* number, *BMI* body-mass index

^a^Available only in Sweden and Denmark

^b^There was no register link to the biological mother or father

^c^Includes the missing or unlikely short (< 22 weeks in Denmark and in Sweden), or long gestational ages (> 45 weeks in Denmark; > 46 weeks in Sweden)

### Main analyses

Among women exposed to RA, 1.8% had a PPD, 0.8% postpartum depression, and 1.0% other PPD, while corresponding figures among the unexposed women were 1.1% for overall PPD, 0.4% for postpartum depression, and 0.6% for other PPD, respectively. Compared to women without RA, women with RA had higher risks of overall PPD (HR = 1.39, 95% CI 1.18 to 1.64), postpartum depression (HR = 1.37, 95% CI 1.07 to 1.77), and other PPD (HR = 1.66, 95% CI 1.34 to 2.05). In analyses stratified by a history of psychiatric disorders before childbirth, we observed increased risks of new-onset overall PPD (HR = 1.52, 95% CI 1.17 to 1.98), postpartum depression (HR = 1.65, 95% CI 1.09 to 2.48), and other PPD (HR = 1.59, 95% CI 1.13 to 2.24) in women with RA relative to their unexposed counterparts. There were no associations between RA and PPD among women with a history of psychiatric disorders before childbirth (Table 2).

**Table 2:** Hazard ratios with 95% confidence intervals for postpartum psychiatric disorders within 90 days after delivery according to rheumatoid arthritis before childbirth, and stratified by a personal history of psychiatric disorders

Outcome	Exposure	No	%	Incidence rate	Crude model	Adjusted model ^a^	*P*-value for the interaction ^b^
				(per 1000 person-years)	HRs (95% CI)	HRs (95% CI)	
**All births**							
Overall PPD	Unexposed to RA	38,790	1.1	45.3	1.00 (Ref.)	1.00 (Ref.)	< 0.001
	Exposed to RA	150	1.8	73.5	1.62 (1.38, 1.90)	1.39 (1.18, 1.64)	
Postpartum depression	Unexposed to RA	15,548	0.4	18	1.00 (Ref.)	1.00 (Ref.)	0.02
	Exposed to RA	63	0.8	30.6	1.69 (1.32, 2.17)	1.37 (1.07, 1.77)	
Other psychiatric disorders postpartum	Unexposed to RA	19,396	0.6	22.6	1.00 (Ref.)	1.00 (Ref.)	0.18
	Exposed to RA	87	1	42.1	1.86 (1.50, 2.30)	1.66 (1.34, 2.05)	
**Births by women without a history of psychiatric disorders**							
Overall PPD	Unexposed to RA	15,374	0.5	19.5	1.00 (Ref.)	1.00 (Ref.)	
	Exposed to RA	57	0.8	32.2	1.66 (1.28, 2.15)	1.52 (1.17, 1.98)	
Postpartum depression	Unexposed to RA	5678	0.2	7.2	1.00 (Ref.)	1.00 (Ref.)	
	Exposed to RA	23	0.3	13	1.81 (1.20, 2.72)	1.65 (1.09, 2.48)	
Other psychiatric disorders postpartum	Unexposed to RA	8675	0.3	11	1.00 (Ref.)	1.00 (Ref.)	
	Exposed to RA	34	0,5	19.2	1.75 (1.25, 2.45)	1.59 (1.13, 2.24)	
**Births by women with a history of psychiatric disorders**							
Overall PPD	Unexposed to RA	23,416	8	346.6	1.00 (Ref.)	1.00 (Ref.)	
	Exposed to RA	93	7.8	339.6	0.98 (0.80, 1.20)	0.88 (0.72, 1.09)	
Postpartum depression	Unexposed to RA	9870	3.4	140.7	1.00 (Ref.)	1.00 (Ref.)	
	Exposed to RA	40	3.4	140.5	1 (0.73, 1.36)	0.84 (0.60, 1.15)	
Other psychiatric disorders postpartum	Unexposed to RA	10,721	3.7	158.7	1.00 (Ref.)	1.00 (Ref.)	
	Exposed to RA	53	4.4	190	1.2 (0.91, 1.57)	1.15 (0.88, 1.51)	

*HRs* hazard ratios, *CI* confidence interval, *No.* number, *PPD* postpartum psychiatric disorders, *RA* rheumatoid arthritis, *Ref.* reference

^a^Adjusted for maternal age at delivery, calendar year of childbirth, parity, maternal highest education levels at delivery, and maternal marital status at delivery

^b^*P*-values correspond to the interaction between RA and a history of psychiatric disorders

**Fig. 1:**
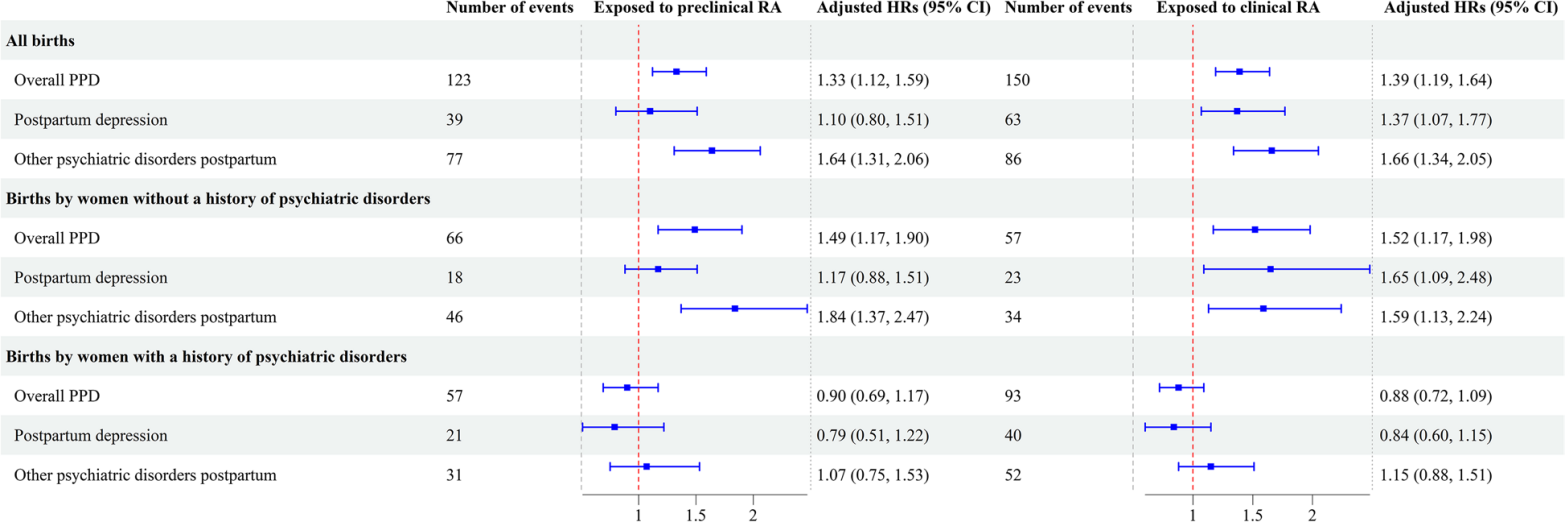
Hazard ratios and 95% confidence intervals for postpartum psychiatric disorders within 90 days after childbirth according to clinical rheumatoid arthritis (rheumatoid arthritis diagnosed before childbirth) and preclinical rheumatoid arthritis (rheumatoid arthritis diagnosed after childbirth). Abbreviations: HRs, hazard ratios; CI, confidence interval; PPD, postpartum psychiatric disorders; RA, rheumatoid arthritis. Adjusted for maternal age at delivery, calendar year of childbirth, parity, maternal highest education levels at delivery, and maternal marital status at delivery. The reference group consists of births by women who unexposed to clinical RA and preclinical RA

Both women with clinical RA and preclinical RA had increased risks of overall PPD and other psychiatric disorders during the first 90 days postpartum (Fig. 1). We found an increased risk of postpartum depression during the first 90 days postpartum only among women with clinical RA. However, when we analyzed postpartum depression during the first 365 days after delivery as the outcome, we observed increased risks of postpartum depression both in women with preclinical RA and clinical RA compared to women unexposed to clinical and preclinical RA (HR = 1.23, 95% CI 1.00 to 1.50 for clinical RA; HR = 1.27, 95% CI 1.03 to 1.56 for preclinical RA, Additional file 1: Fig. S2). In analyses stratified by a history of psychiatric disorders, preclinical RA was associated with increased risks of overall PPD, postpartum depression, and other PPD among women without a history of psychiatric disorders, but not among those with such a history (Fig. 1).

### Sensitivity analyses

Further adjustment for maternal BMI in early pregnancy, country of origin, and a family history of psychiatric disorders among those with such data did not change the results substantially (Additional file 1: Table S5 and S6). In the sibling analyses, the association between RA and PPD disappeared, but we observed a trend towards an association between RA and an increased risk of new-onset postpartum depression among women without a psychiatric history (Additional file 1: Table S7). Adjusting for SGA, preterm birth, stillbirth, pre-eclampsia, or placental abruption one by one did not substantially alter the association between RA and the risk of PPD (Additional file 1: Table S8). The point estimates corresponding to the association between RA and PPD, postpartum depression, and other PPD among women without a history of psychiatric disorders before childbirth were somewhat higher when we restricted the analyses to primiparous women compared with those observed in the main analyses (Additional file 1: Table S9). The association of RA with the risk of new-onset PPD was stronger in diabetic women than among non-diabetic women (*P*-value for interaction = 0.02, Additional file 1: Table S10). The associations did not differ between women with and without pre-eclampsia.

After performing a PSM analysis, the exposed (*N* = 8407) and unexposed (*N* = 8407) cohorts were comparable in terms of most of the baseline characteristics at birth (Additional file 1: Table S11). The patterns of the associations were similar to those observed in the main analyses (Additional file 1: Table S12). In analyses restricted to women with a history of psychiatric disorders, we found that RA tended to be associated with an increased risk of PPD in women without a history of psychiatric medications, but not in those with a history of psychiatric medications (Additional file 1: Table S13). When we restricted the definition of preclinical RA to having the first diagnosis of RA 1 year after childbirth (Additional file 1: Fig. S3) or performed analyses with our secondary outcome, i.e., PPD in the first 365 days, results were similar to those obtained in the primary analyses (Additional file 1: Fig. S4).

## Discussion

We found that RA was associated with increased risks of new-onset PPDs, including postpartum depression, and other PPD among women without a history of psychiatric disorders, but not among women with a history of psychiatric disorders. We also observed largely similar associations between preclinical RA (i.e., RA diagnosed after childbirth) and PPD to those corresponding to clinical RA.

Several population-based cohort studies examined the associations of immune-related disorders, e.g., pre-eclampsia, autoimmune thyroid disorders, inflammatory bowel disease, and atopic diseases, with PPD risk [5, 32–34]. Only Feldman and associates studied the association between some type of rheumatic disease and the risk of PPD; they found that women with juvenile idiopathic arthritis had a lower risk of postpartum depression than their unexposed counterparts [14]. Earlier studies regarding the association between different types of rheumatic disease and the risk of psychiatric disorders in general population samples have also been inconsistent [35–37]. Previous studies regarding immune-related disorders and PPD risk generally excluded multiparous women or women with a history of psychiatric disorders before delivery. We are the first to examine the associations between RA before childbirth and PPD risk, as well as to stratify analyses concerning immune-related diseases and PPD risk by women’s history of psychiatric disorders.

The lack of an association among women with a history of psychiatric disorders in our study may be somewhat unexpected, given the well-documented increased risk of postpartum psychiatric relapse in this group [38]. One possible explanation is that the diagnosis of PPD among women with a history of psychiatric disorders is very likely to be a regular visit for their pre-existing psychiatric disorder. If the PPD only accounts for a minor part of all psychiatric disorders in women with a history of psychiatric disorder, the competing risk from other psychiatric disorders may have attenuated the association between RA and PPD in this group. Second, in anticipation of their potentially difficult postpartum period arising from a high psychiatric vulnerability, increased RA activity, sleep deprivation, and the psychological demands of caring for a newborn, women with a history of psychiatric disorders may be more likely to receive prophylactic antidepressants or anti-psychotic medications. When we further performed stratified analyses according to their prescriptions of psychiatric medications, we found a trend towards an association between RA and an increased risk of PPD only among women without a history of psychiatric medications. This result partially supports that intake of psychiatric medications might contribute to the explanation of our lack of an association between RA and PPD among women with a history of psychiatric disorders. However, we did not have information on psychiatric medication for a sufficiently large part of the cohort to detect significant differences in this respect. Third, since only psychiatric disorders diagnosed in specialized outpatient care and inpatient care are included in the Nordic registers, there is likely to be an underreporting with respect to the history of psychiatric disorders [25]; this misclassification may have limited statistical power to detect modest associations between RA and PPD in this group.

A concern to address in studies regarding the association between RA and the risk of psychiatric disorders is the separation of the inflammation-related effects of RA from the effects of RA treatment and care. We tried to disentangle the role of treatment and care for RA from the RA per se by examining the risk of PPD among women with preclinical RA, given that the preclinical RA involves increased inflammation typically associated with active RA, but without severe symptoms, taking any treatment and care yet [23]. Increased risks of postpartum depression during the first 365 days after birth were observed irrespective of whether the women were exposed to clinical or preclinical RA, although the risks were less consistent for postpartum depression during the first 90 days due to limited power in the group with preclinical RA. These findings may point towards an effect of the disease rather than the effects of RA-specific treatment or care or the effects of limitations of life activities resulting from severe RA symptoms. Another potential explanation for this finding is that women with PPD might also have a higher overall risk of subsequent RA, indicating a bidirectional association between RA and PPD, as suggested in a previous study [39, 40].

The potential biological mechanisms underlying the association between RA and PPD are unclear. The role of inflammation in the association between RA and depression outside the pregnancy is well-known [8] and may also account for the association between RA and postpartum depression. Upregulation of major proinflammatory cytokines, such as inter-leukin-1β and tumor necrosis factor α, play an important role in the pathogenesis of RA [12]. Similarly, the activation of the inflammatory response is involved in the pathophysiology of postpartum depression [41]. Potential mechanisms linking inflammation due to RA to changes in the activity of the central nervous system have been identified in several animal models [42–44]. Immune-mediated inflammation can modulate neurotransmission, neurogenesis, neuroendocrine activity, and neuroplasticity and affect corticolimbic circuits involved in emotion and stress regulation [8]. Additionally, the cytokines could induce alterations in HPA axis and glucocorticoid function, which are well known to be associated with PPD [8]. We found that in women without a history of psychiatric disorders, the association between RA and overall PPD was strengthened by the presence of diabetes. Given that alterations in dysregulation of the HPA axis and cytokine-mediated inflammatory responses can be induced by either RA or diabetes [45, 46], an interaction between shared mechanistic pathways might be biologically plausible. Further studies are needed to explore the mechanisms underlying the observed associations.

The main strength of our study is the use of large-scale register-based data from Denmark, Finland, and Sweden. The study includes nearly all births in these countries during up to two decades and prospectively collects information on exposure, outcome, and covariates, which reduces the risks of selection and recall bias. Some limitations of the study need to be taken into consideration when interpreting our findings. First, as our study was register-based, we could identify only the most severe psychiatric disorders, but not patients who sought care only in primary care [25], and thus, we may have underestimated the incidence of PPD and that of personal and family history of psychiatric disorders. Validation studies of the RA diagnoses in the National Patient Register reported a positive predictive value of 59% in Danish data [47], 82% in Finnish data [48], and 91% in Swedish data [49]. However, these potential misclassifications of exposure and outcome would most likely be non-differential and thus might bias the risk estimates towards the null. Second, although we adjusted for a wide range of potential confounders, residual confounding cannot be ruled out, given that some important information on treatment of RA, RA disease activity, and available informal care and social support of RA was not available. However, we expect the potential impact of RA treatment and care to be modest because of the similar associations we observed between preclinical RA and PPD. Third, although we have a large cohort from three countries, the number of events is small in some sub-analyses. Fourth, we used sibling design to control for unmeasured genetic and environmental factors shared by families and observed a trend towards an increased risk of postpartum depression in women with RA. However, caution is needed when interpreting these findings given the very limited number of siblings discordant for exposure and outcome. Additionally, sibling comparisons are, by design, associated with some limitations, such as residual confounding not shared by siblings [50].

## Conclusions

In this large population-based cohort study from three Nordic countries, we found that among women without a history of psychiatric disorders, those with RA had a higher risk of PPD, especially postpartum depression, than their unexposed counterparts. There were no associations between RA and PPD among women with a history of psychiatric disorders. If our findings are confirmed in future studies, new mothers with RA may benefit from increased surveillance for new-onset psychiatric disorders postpartum.

## Supplementary Information

**Additional file 1: Table S1.** Description of the registers included in the study. **Table S2.** The International Classification of Diseases codes used to define medical conditions. **Table S3.** Incidence proportion and incidence rates of postpartum psychiatric disorders within 90 days after childbirth in the study cohort. **Table S4.** Characteristics of the study population according to clinical rheumatoid arthritis and preclinical rheumatoid arthritis. **Table S5.** Hazard ratios and 95% confidence intervals for postpartum psychiatric disorders within 90 days after childbirth according to rheumatoid arthritis before childbirth, after additional adjustment for maternal body-mass index during early pregnancy. **Table S6.** Hazard ratios and 95% confidence intervals for postpartum psychiatric disorders within 90 days after childbirth according to rheumatoid arthritis before childbirth, after additional adjustment for maternal country origin and family history of psychiatric disorders. **Table S7.** Hazard ratios and 95% confidence intervals for postpartum psychiatric disorders within 90 days after childbirth according to rheumatoid arthritis before childbirth in a sibling design. **Table S8.** Hazard ratios and 95% confidence intervals for postpartum psychiatric disorders within 90 days after childbirth according to rheumatoid arthritis before childbirth after adjusting for potential explanatory factors. **Table S9.** Hazard ratios and 95% confidence intervals for postpartum psychiatric disorders within 90 days after childbirth according to rheumatoid arthritis before childbirth, in analyses restricted to primiparous women. **Table S10.** Hazard ratios and 95% confidence intervals for postpartum psychiatric disorders within 90 days after childbirth according to rheumatoid arthritis before childbirth in stratified analyses. **Table S11.** Baseline characteristics of the propensity score matching sub-cohort. **Table S12.** Hazard ratios and 95% confidence intervals for postpartum psychiatric disorders within 90 days after childbirth according to rheumatoid arthritis before childbirth in the propensity-matched sub-cohort. **Table S13.** Hazard ratios and 95% confidence intervals for the associations between rheumatoid arthritis before childbirth and postpartum psychiatric disorders within 90 days after childbirth among women with a personal history of psychiatric disorders, further stratified by previous use of psychiatric medication. **Fig S1.** The time trend of the postpartum psychiatric disorder rates over the study period in the exposed and the unexposed group. **Fig S2.** Hazard ratios and 95% confidence intervals for postpartum psychiatric disorders within 365 days after childbirth according to clinical rheumatoid arthritis (rheumatoid arthritis diagnosed before childbirth) and preclinical rheumatoid arthritis (rheumatoid arthritis diagnosed after childbirth). **Fig S3.** Hazard ratios and 95% confidence intervals for postpartum psychiatric disorders within 365 days after childbirth according to clinical rheumatoid arthritis (rheumatoid arthritis diagnosed before childbirth) and preclinical rheumatoid arthritis (rheumatoid arthritis diagnosed in the first year after childbirth). **Fig S4.** Hazard ratios and 95% confidence intervals for the associations between rheumatoid arthritis before childbirth and postpartum psychiatric disorders within the first 365 days after delivery, stratified by a personal history of psychiatric disorders.

## Data Availability

Data used for this study are not openly available because the law and the ethical permit does not allow to share them. The data were analyzed on a secure server at Statistics Denmark run by Aarhus University.

## Abbreviations

BMI: Body mass index
CIs: Confidence intervals
HRs: Hazard ratios
ICD: International Statistical Classification of Diseases and Related Health Problems
PPD: Postpartum psychiatric disorders
PSM: Propensity score-matched
RA: Rheumatoid arthritis
SGA: Small for gestational age
