# Supplementary material for: Rheumatoid arthritis and the risk of postpartum psychiatric disorders: a Nordic population-based cohort study
Source: BMC Med. 2023 Apr 3;21:126. doi: 10.1186/s12916-023-02837-3 (PMC10071633; doi:10.1186/s12916-023-02837-3)
Supplement: Supplementary file 1 — Additional file 1: Table S1. Description of the registers included in the study. Table S2. The International Classification of Diseases codes used to define medical conditions. Table S3. Incidence proportion and incidence rates of postpartum psychiatric disorders within 90 days after childbirth in the study cohort. Table S4. Characteristics of the study population according to clinical rheumatoid arthritis and preclinical rheumatoid arthritis. Table S5. Hazard ratios and 95% confidence intervals for postpartum psychiatric disorders within 90 days after childbirth according to rheumatoid arthritis before childbirth, after additional adjustment for maternal body-mass index during early pregnancy. Table S6. Hazard ratios and 95% confidence intervals for postpartum psychiatric disorders within 90 days after childbirth according to rheumatoid arthritis before childbirth, after additional adjustment for maternal country origin and family history of psychiatric disorders. Table S7. Hazard ratios and 95% confidence intervals for postpartum psychiatric disorders within 90 days after childbirth according to rheumatoid arthritis before childbirth in a sibling design. Table S8. Hazard ratios and 95% confidence intervals for postpartum psychiatric disorders within 90 days after childbirth according to rheumatoid arthritis before childbirth after adjusting for potential explanatory factors. Table S9. Hazard ratios and 95% confidence intervals for postpartum psychiatric disorders within 90 days after childbirth according to rheumatoid arthritis before childbirth, in analyses restricted to primiparous women. Table S10. Hazard ratios and 95% confidence intervals for postpartum psychiatric disorders within 90 days after childbirth according to rheumatoid arthritis before childbirth in stratified analyses. Table S11. Baseline characteristics of the propensity score matching sub-cohort. Table S12. Hazard ratios and 95% confidence intervals for postpartum psychiatric disorders within 90 [file 12916_2023_2837_MOESM1_ESM.docx]

**Supplementary Materials**

**Rheumatoid arthritis and the risk of postpartum psychiatric disorders: A Nordic population-based cohort study**

Min Luan ^1,2,3,*^, Fen Yang ^2^, Maohua Miao ^1^, Wei Yuan ^1^, Mika Gissler ^4,5,6^, Elizabeth V Arkema ^7^, Donghao Lu ^8^, Jiong Li ^9^, Krisztina D. László ^2,10^

**Affiliations：**

^1^ NHC Key Laboratory of Reproduction Regulation (Shanghai Institute for Biomedical and Pharmaceutical Technologies), Fudan University, Shanghai, China

^2^ Department of Global Public Health, Karolinska Institutet, Stockholm, Sweden

^3^ Clinical Research Center, Shanghai Sixth People’s Hospital Affiliated to Shanghai Jiao Tong University School of Medicine, Shanghai, China

^4^ Department of Knowledge Brokers, Finnish Institute for Health and Welfare, Helsinki, Finland

^5^ Academic Primary Health Care Centre, Region Stockholm, Stockholm, Sweden

^6^ Department of Molecular Medicine and Surgery, Karolinska Institutet, Stockholm, Sweden

^7^ Clinical Epidemiology Division, Department of Medicine Solna, Karolinska Institutet, Stockholm, Sweden

^8^ Unit of Integrative Epidemiology, Institute of Environmental Medicine, Karolinska Institutet, Stockholm, Sweden

^9^ Department of Clinical Medicine-Department of Clinical Epidemiology, Aarhus University, Denmark

^10^ Department of Public Health and Caring Sciences, Uppsala University, Uppsala, Sweden

***Correspondence to**: Min Luan, Department of Global Public Health, Karolinska Institutet, Tomtebodavägen 18A, 171 77 Stockholm, Sweden.

E-mail: min.luan.2@ki.se

**Contents**

**Table S1.** Description of the registers included in the study

**Table S2.** The International Classification of Diseases codes used to define medical conditions

**Table S3.** Incidence proportion and incidence rates of postpartum psychiatric disorders within 90 days after childbirth in the study cohort

**Table S4.** Characteristics of the study population according to clinical rheumatoid arthritis and preclinical rheumatoid arthritis

**Table S5.** Hazard ratios and 95% confidence intervals for postpartum psychiatric disorders within 90 days after childbirth according to rheumatoid arthritis before childbirth, after additional adjustment for maternal body-mass index during early pregnancy

**Table S6.** Hazard ratios and 95% confidence intervals for postpartum psychiatric disorders within 90 days after childbirth according to rheumatoid arthritis before childbirth, after additional adjustment for maternal country origin and family history of psychiatric disorders

**Table S7.** Hazard ratios and 95% confidence intervals for postpartum psychiatric disorders within 90 days after childbirth according to rheumatoid arthritis before childbirth in a sibling design (n =1,839,049)

**Table S8.** Hazard ratios and 95% confidence intervals for postpartum psychiatric disorders within 90 days after childbirth according to rheumatoid arthritis before childbirth after adjusting for potential explanatory factors

**Table S9.** Hazard ratios and 95% confidence intervals for postpartum psychiatric disorders within 90 days after childbirth according to rheumatoid arthritis before childbirth, in analyses restricted to primiparous women (n = 1,444,760)

**Table S10.** Hazard ratios and 95% confidence intervals for postpartum psychiatric disorders within 90 days after childbirth according to rheumatoid arthritis before childbirth in stratified analyses

**Table S11.** Baseline characteristics of the propensity score matching sub-cohort (N = 16,814)

**Table S12.** Hazard ratios and 95% confidence intervals for postpartum psychiatric disorders within 90 days after childbirth according to rheumatoid arthritis before childbirth in the propensity-matched sub-cohort

**Table S13.** Hazard ratios and 95% confidence intervals for the associations between rheumatoid arthritis before childbirth and postpartum psychiatric disorders within 90 days after childbirth among women with a personal history of psychiatric disorders, further stratified by previous use of psychiatric medication

**Figure S1.** The time trend of the postpartum psychiatric disorder rates over the study period in the exposed and the unexposed group

**Figure S2.** Hazard ratios and 95% confidence intervals for postpartum psychiatric disorders within 365 days after childbirth according to clinical rheumatoid arthritis (rheumatoid arthritis diagnosed before childbirth) and preclinical rheumatoid arthritis (rheumatoid arthritis diagnosed after childbirth)

**Figure S3.** Hazard ratios and 95% confidence intervals for postpartum psychiatric disorders within 365 days after childbirth according to clinical rheumatoid arthritis (rheumatoid arthritis diagnosed before childbirth) and preclinical rheumatoid arthritis (rheumatoid arthritis diagnosed in the first year after childbirth)

**Figure S4.** Hazard ratios and 95% confidence intervals for the associations between rheumatoid arthritis before childbirth and postpartum psychiatric disorders within the first 365 days after delivery, stratified by a personal history of psychiatric disorders

**Table S1.** Description of the registers included in the study

| **Data source** | **The Information** |  |
| --- | --- | --- |
| Denmark |  |  |
| National Hospital Register | The register contains information on dates of admission and diagnoses in inpatient hospitalizations since 1977. Diagnoses from hospital outpatient care have been included in this register since 1995. All psychiatric inpatient, psychiatric specialized outpatient and emergency contacts have been included in the register since 1995. |  |
|  |  |  |
| Medical Birth Register | The Medical Birth Register contains information on maternal age at delivery, parity, the date of birth, gestational age, birth weight, stillbirth, and singleton/multiple pregnancy since 1973, and maternal body-mass index in early pregnancy since 2004. |  |
| Danish Civil Registration System | The register includes information on the individual personal identification number, date and place of birth, marital status, date of immigration, vital status, as well as family relationships (e.g., identity of parents). |  |
| The Integrated Database for Labor Market Research | We extracted information on education and country of origin. |  |
| Psychiatric Central Research Register | The registry was established as a nationwide register in 1969 and includes information on psychiatric hospitalizations in Denmark and includes outpatient visits since 1995. It covers information on diagnostic codes, admission, and discharge dates. |  |
|  |  |  |
| **Finland** |  |  |
| Hospital Discharge Register | The register covers all inpatient hospital diagnoses since 1969, and outpatient diagnoses since 1998. We had data from this register since 1987. |  |
|  |  |  |
| Medical Birth Register | The Finnish Medical Birth Register, established in 1987, contains information on all births in Finland. We extracted information from this register on maternal age at delivery, parity, gestational age, birth weight, and maternal marital status at delivery. |  |
|  |  |  |
| Education Register | The register contains data on the highest educational level. We had data from this register since 1987. |  |
| Cause of Death Register | The register contains data on all deaths and their causes. Information on the date of death was available since 1976. |  |
| **Sweden** |  |  |
| National Patient Register | The Swedish Patient Register (established in parts of Sweden in 1964; nationwide since 1987) contains information on dates of hospital admission and discharge, and diagnoses on discharge classified according to the ICD. Since 2001, this registry has also included information of outpatient hospital visits. |  |
| Medical Birth Register | The Medical Birth Register, established in 1973, provides medical data on all births in Sweden. Information on complications during pregnancy and delivery, maternal age, maternal height and weight, parity, gestational age, birth weight, date of birth, sex, singleton status, stillbirth and maternal country of origin was recorded. |  |
| Education Register | The register records information on highest level of completed formal education. |  |
| Multi-Generation Register | The Multi‐Generation Register contains information on all residents in Sweden who were born in 1932 or later and on their parents. Information on biological relationships and siblings for all individuals residing in Sweden was recorded since 1961. |  |
|  |  |  |
| Total Population Register | The Total Population Register is available for every year since 1968 and includes among others information on marital status, and date of immigration. |  |
| Cause of Death Register | We extracted information on the date of death. |  |

Abbreviations: ICD, International Statistical Classification of Diseases and Related Health Problems.

**Table S2**. The International Classification of Diseases codes used to define medical conditions

| Medical conditions | **ICD codes** | | | | | | | | |
| --- | --- | --- | --- | --- | --- | --- | --- | --- | --- |
|  | **Denmark** | |  | **Sweden** | | |  | **Finland** | |
|  | **ICD-8** | **ICD-10** |  | **ICD-8** | **ICD-9** | **ICD-10** |  | **ICD-9** | **ICD-10** |
| RA | 712.19, 712.39, 712.59, 712.09 | M05, M06 |  | 712.10, 712.38, 712.39, 712.50,712.00 | 714A, 714B, 714C, 714D, 714W | M05, M06 |  | 7140A, 7140B, 7141A, 7142X, 7143A | M05, M06 |
| Psychiatric disorders | 290-315 | F00-F99 |  | 290-315 | 290-319 | F00-F99 |  | 290-319 | F00-F99 |
| PPD |  |  |  |  |  |  |  |  |  |
| Overall PPD | - | F00-F99 |  | - | - | F00-F99 |  | - | F00-F99 |
| Postpartum depression | - | F32, F33, F34, F38, F39, F53.0 |  | - | - | F32, F33, F34, F38, F39, F53.0 |  | - | F32, F33, F34, F38, F39, F53.0 |
| Diabetes | 249, 250 | E10-E14, O24 |  | 250 | 250, 648A, 648W | E10-E14, O24 |  | 250, 6480A, 6488A | E10-E14, O24 |
| Preeclampsia | - | O14, O15 |  | - | - | O14, O15 |  | - | O14, O15 |
| Placental abruption | - | O45 |  | - | - | O45 |  | - | O45 |

Abbreviations: ICD, International Statistical Classification of Diseases and Related Health Problems; RA, Rheumatoid arthritis; PPD, postpartum psychiatric disorders.

Note: In Denmark, ICD-8 was used during 1967-1993 and ICD-10 since 1994, while in Sweden ICD-8 during 1968-1986, ICD-9 during 1987-1996, and ICD-10 since 1997, and in Finland ICD-9 during 1987-1995 and ICD-10 since 1996.

**Table S3.** Incidence proportion and incidence rates of postpartum psychiatric disorders within 90 days after childbirth in the study cohort

|  | No. (%) | Incidence rates per 1000 person-years |
| --- | --- | --- |
| **All births (n=3,516,849)** | | |
| Overall PPD | 38940 (1.11) | 45.32 |
| Postpartum depression | 15611 (0.44) | 18.08 |
| Other psychiatric disorders postpartum | 19482 (0.55) | 22.67 |
| **Births by women without a history of psychiatric disorders (n=3,223,478, 91.66%)** | | |
| Overall PPD | 15431 (0.84) | 19.50 |
| Postpartum depression | 5701 (0.18) | 7.19 |
| Other psychiatric disorders postpartum | 8709 (0.27) | 11.00 |
| **Births by women with a history of psychiatric disorders (n=293,371, 8.34%)** | | |
| Overall PPD | 23509 (8.01) | 346.52 |
| Postpartum depression | 9910 (3.38) | 140.72 |
| Other psychiatric Disorders postpartum | 10773 (3.67) | 158.79 |

Abbreviations: PPD, postpartum psychiatric disorders; No. number.

**Table S4.** Characteristics of the study population according to clinical rheumatoid arthritis and preclinical rheumatoid arthritis

|  | Births of women | | | | | | | |  |
| --- | --- | --- | --- | --- | --- | --- | --- | --- | --- |
|  | With clinical RA (N = 8,406) | |  | With preclinical RA (N = 11,951) | |  | Without clinical or  preclinical RA  (N = 3,496,492) | |  |
|  |  |  |  |  |  |  |  |  |  |
|  | No. | % |  | No. | % |  | No. | % |  |
| **Maternal characteristics** | | | | | |  |  |  |  |
| Study country | | | | | |  |  |  |  |
| Denmark | 4,297 | 51.12 |  | 7,641 | 63.94 |  | 1,284,344 | 36.73 |  |
| Finland | 215 | 2.56 |  | 322 | 2.69 |  | 912,048 | 26.09 |  |
| Sweden | 3,895 | 46.32 |  | 3,988 | 33.37 |  | 1,300,099 | 37.18 |  |
| Maternal age at delivery (years) | | | | | |  |  |  |  |
| <25 | 735 | 8.74 |  | 1,609 | 13.46 |  | 537,476 | 15.37 |  |
| 25-29 | 2,271 | 27.02 |  | 3,730 | 31.21 |  | 1,112,473 | 31.82 |  |
| 30-34 | 3,163 | 37.62 |  | 4,159 | 34.80 |  | 1,184,772 | 33.88 |  |
| ≥35 | 2,238 | 26.62 |  | 2,453 | 20.53 |  | 661,770 | 18.93 |  |
| Maternal country of origin ^a^ | | | | | |  |  |  |  |
| Denmark/Sweden | 4,262 | 52.03 |  | 5,009 | 43.07 |  | 1,489,115 | 57.62 |  |
| Other countries | 3,929 | 47.96 |  | 6,619 | 56.92 |  | 1,093,175 | 42.30 |  |
| Unknown | <5 | 0.01 |  | <5 | 0.01 |  | 2,153 | 0.08 |  |
| Maternal BMI in early pregnancy (kg/m^2^) ^a^ | | | | | |  |  |  |  |
| <18.5 | 204 | 2.49 |  | 155 | 1.33 |  | 57,686 | 2.23 |  |
| 18.5-24.9 | 3,728 | 45.51 |  | 3,008 | 25.87 |  | 1,081,394 | 41.84 |  |
| 25.0-29.9 | 1,654 | 20.19 |  | 1,599 | 13.75 |  | 473,873 | 18.34 |  |
| ≥30.0 | 876 | 10.69 |  | 836 | 7.19 |  | 234,297 | 9.07 |  |
| Unknown | 1,730 | 21.12 |  | 6,031 | 51.86 |  | 737,193 | 28.52 |  |
| Marital status at delivery | | | | | |  |  |  |  |
| Single, widowed, or divorced | 4,710 | 56.03 |  | 6,194 | 51.83 |  | 1,755,503 | 50.21 |  |
| Married or in registered partnership | 3,690 | 43.89 |  | 5,740 | 48.03 |  | 1,706,874 | 48.82 |  |
| Unknown | 7 | 0.08 |  | 17 | 0.14 |  | 34,114 | 0.97 |  |
| Maternal highest education at delivery | | | | | |  |  |  |  |
| Primary and lower secondary | 1,226 | 14.58 |  | 2,628 | 21.99 |  | 789,421 | 22.58 |  |
| Upper secondary | 3,449 | 41.03 |  | 5,618 | 47.01 |  | 1,229,672 | 35.17 |  |
| University | 3,668 | 43.63 |  | 3,561 | 29.80 |  | 1,331,332 | 38.07 |  |
| Unknown | 64 | 0.76 |  | 144 | 1.20 |  | 146,066 | 4.18 |  |
| Maternal smoking in early pregnancy | | | | | |  |  |  |  |
| No | 7,122 | 84.72 |  | 8,916 | 74.60 |  | 2,946,071 | 84.26 |  |
| Yes | 957 | 11.38 |  | 2,467 | 20.64 |  | 418,720 | 11.97 |  |
| Unknown | 328 | 3.90 |  | 568 | 4.75 |  | 131,700 | 3.77 |  |
| Parity | | | | | |  |  |  |  |
| Primiparous | 3,533 | 42.02 |  | 4,777 | 39.97 |  | 1,436,450 | 41.08 |  |
| Multiparous | 4,874 | 57.98 |  | 7174 | 60.03 |  | 2,059,173 | 58.89 |  |
| Unknown | 0 | 0.00 |  | 0 | 0.00 |  | 868 | 0.03 |  |
| Psychiatric disorders before childbirth | | | | | |  |  |  |  |
| No | 7,221 | 85.89 |  | 10,998 | 92.03 |  | 3,205,259 | 91.67 |  |
| Yes | 1,186 | 14.11 |  | 953 | 7.97 |  | 291,232 | 8.33 |  |
| Family history of psychiatric disorders ^a^ | | | | |  |  |  |  |  |
| No | 6,631 | 80.94 |  | 8,973 | 77.16 |  | 1,951,491 | 75.51 |  |
| Yes | 1,040 | 12.70 |  | 1,251 | 10.76 |  | 239,099 | 9.25 |  |
| Unknown ^b^ | 521 | 6.36 |  | 1,405 | 12.08 |  | 393,953 | 15.24 |  |
| Diabetes | | | | | |  |  |  |  |
| No | 7,984 | 94.97 |  | 11,513 | 96.34 |  | 3,338,294 | 95.48 |  |
| Yes | 423 | 5.03 |  | 438 | 3.66 |  | 158,197 | 4.52 |  |
| Pre-eclampsia | | | | | |  |  |  |  |
| No | 7,996 | 95.11 |  | 11,518 | 96.38 |  | 3,398,538 | 97.20 |  |
| Yes | 411 | 4.89 |  | 433 | 3.62 |  | 97,953 | 2.80 |  |
| Placental abruption | | | | | |  |  |  |  |
| No | 8,363 | 99.48 |  | 11,889 | 99.48 |  | 3,484,398 | 99.65 |  |
| Yes | 44 | 0.52 |  | 62 | 0.52 |  | 12,093 | 0.35 |  |
| **Characteristics of the birth** | | | | | |  |  |  |  |
| Calendar year of birth | | | | | |  |  |  |  |
| 1995-2000 | 759 | 9.03 |  | 4,141 | 34.65 |  | 562,049 | 16.08 |  |
| 2001-2005 | 2,077 | 24.70 |  | 4,594 | 38.44 |  | 1,043,656 | 29.85 |  |
| 2006-2010 | 3,017 | 35.89 |  | 2,546 | 21.30 |  | 1,116,891 | 31.94 |  |
| 2011-2015 | 2,554 | 30.38 |  | 670 | 5.61 |  | 773,895 | 22.13 |  |
| Small for gestational age | | | | | |  |  |  |  |
| No | 7,280 | 86.60 |  | 10,364 | 86.72 |  | 3,104,771 | 88.80 |  |
| Yes | 1,009 | 12.00 |  | 1,348 | 11.28 |  | 344,653 | 9.86 |  |
| Unknown | 118 | 1.40 |  | 239 | 2.00 |  | 47,067 | 1.34 |  |
| Preterm birth (gestational < 37 weeks) | | | | | |  |  |  |  |
| No | 7,675 | 91.30 |  | 11,003 | 92.07 |  | 3,296,205 | 94.27 |  |
| Yes | 667 | 7.93 |  | 781 | 6.53 |  | 169,689 | 4.85 |  |
| Unknown ^c^ | 65 | 0.77 |  | 167 | 1.40 |  | 30,597 | 0.88 |  |
| Stillbirth | | | | | |  |  |  |  |
| No | 8,367 | 99.52 |  | 11,908 | 99.64 |  | 3,487,246 | 99.74 |  |
| Yes | 40 | 0.48 |  | 43 | 0.36 |  | 9,245 | 0.26 |  |

Abbreviations: RA, rheumatoid arthritis; No, number; BMI, body-mass index.

^a^ Available only in Sweden and Denmark.

^b^ There was no register link to the biological mother or father.

^c^ Includes the missing or unlikely short (<22 weeks in Denmark and in Sweden), or long gestational age (>45 weeks in Denmark; >46 weeks in Sweden).

**Table S5.** Hazard ratios and 95% confidence intervals for postpartum psychiatric disorders within 90 days after childbirth according to rheumatoid arthritis before childbirth, after additional adjustment for maternal body-mass index during early pregnancy

|  | Number of events / person-years in women with RA | Adjusted HRs ^a^ (95% CI) |
| --- | --- | --- |
| **All births ^b^ (n =1,819,522)** | | |
| Overall PPD | 123/1559 | 1.27 (1.06, 1.51) |
| Postpartum depression | 52/1574 | 1.30 (0.99, 1.70) |
| Other psychiatric disorders postpartum | 70/1559 | 1.43 (1.13, 1.81) |
| **Births by women without a history of psychiatric disorders (n =1,630,242)** | | |
| Overall PPD | 47/1331 | 1.45 (1.09, 1.93) |
| Postpartum depression | 20/1336 | 1.61 (1.04, 2.51) |
| Other psychiatric disorders postpartum | 27/1331 | 1.45 (0.99, 2.12) |
| **Births by women with a history of psychiatric disorders (n =189,280)** | | |
| Overall PPD | 76/228 | 0.85 (0.68, 1.07) |
| Postpartum depression | 32/238 | 0.84 (0.59, 1.19) |
| Other psychiatric disorders postpartum | 43/228 | 1.04 (0.77, 1.40) |

Abbreviations: HRs, hazard ratios; CI, confidence interval; PPD, postpartum psychiatric disorders; RA, rheumatoid arthritis.

^a^ Adjusted for maternal age at delivery, calendar year of childbirth, parity, maternal highest education levels at delivery, maternal marital status at delivery, and maternal body-mass index during early pregnancy.

^b^ Study participants with missing information on body-mass index were excluded.

**Table S6.** Hazard ratios and 95% confidence intervals for postpartum psychiatric disorders within 90 days after childbirth according to rheumatoid arthritis before childbirth, after additional adjustment for maternal country origin and family history of psychiatric disorders

|  | Number of events / person-years in women with RA | Adjusted HRs ^a^ (95% CI) |
| --- | --- | --- |
| **All births ^b^ (n = 2,204,258)** | | |
| Overall PPD | 130/1862 | 1.24 (1.04, 1.47) |
| Postpartum depression | 52/1878 | 1.24 (0.94, 1.62) |
| Other psychiatric disorders postpartum | 77/1862 | 1.42 (1.14, 1.78) |
| **Births by women without a history of psychiatric disorders (n = 1,998,782)** | | |
| Overall PPD | 50/1610 | 1.23 (0.93, 1.63) |
| Postpartum depression | 21/1616 | 1.40 (0.91, 2.16) |
| Other psychiatric Disorders postpartum | 29/1610 | 1.23 (0.86, 1.78) |
| **Births by women with a history of psychiatric disorders (n = 205,476)** | | |
| Overall PPD | 80/252 | 0.90 (0.72, 1.12) |
| Postpartum depression | 31/262 | 0.81 (0.57, 1.15) |
| Other psychiatric disorders postpartum | 48/252 | 1.16 (0.87, 1.54) |

Abbreviations: HRs, hazard ratios; CI, confidence interval; PPD, postpartum psychiatric disorders; RA, rheumatoid arthritis.

^a^ Adjusted for maternal age at delivery, calendar year of childbirth, parity, maternal highest education levels at delivery, maternal marital status at delivery, maternal country origin and family history of psychiatric disorders (biological parents).

^b^ Study participants with missing information on maternal country origin and family history of psychiatric disorders were excluded.

**Table S7.** Hazard ratios and 95% confidence intervals for postpartum psychiatric disorders within 90 days after childbirth according to rheumatoid arthritis before childbirth in a sibling design (n =1,839,049)

|  |  | Case numbers | Incidence rate (per 1000 person-years) | Crude HRs (95% CI) | Adjusted HRs ^a^ (95% CI) |
| --- | --- | --- | --- | --- | --- |
| **All births ^b^ (n = 1,839,049)** | | | | | |
| Overall PPD | Unexposed to RA | 19757 | 44.11 | 1.00 (Ref.) | 1.00 (Ref.) |
|  | Exposed to RA | 88 | 57.23 | 1.02 (0.69, 1.50) | 0.89 (0.60, 1.32) |
| Postpartum depression | Unexposed to RA | 7863 | 17.47 | 1.00 (Ref.) | 1.00 (Ref.) |
|  | Exposed to RA | 40 | 25.84 | 1.27 (0.69, 2.31) | 1.03 (0.55, 1.91) |
| Other psychiatric disorders postpartum | Unexposed to RA | 10403 | 23.23 | 1.00 (Ref.) | 1.00 (Ref.) |
|  | Exposed to RA | 48 | 31.22 | 1.00 (0.60, 1.68) | 0.93 (0.55, 1.57) |
| **Births by women without a history of psychiatric disorders (n = 1,672,743)** | | | | | |
| Overall PPD | Unexposed to RA | 8457 | 20.66 | 1.00 (Ref.) | 1.00 (Ref.) |
|  | Exposed to RA | 34 | 25.36 | 1.55 (0.82, 2.95) | 1.09 (0.56, 2.12) |
| Postpartum depression | Unexposed to RA | 3005 | 7.33 | 1.00 (Ref.) | 1.00 (Ref.) |
|  | Exposed to RA | 16 | 11.9 | 3.18 (0.99, 10.21) | 2.14 (0.64, 7.14) |
| Other psychiatric disorders postpartum | Unexposed to RA | 5013 | 12.23 | 1.00 (Ref.) | 1.00 (Ref.) |
|  | Exposed to RA | 18 | 13.42 | 1.19 (0.53, 2.69) | 0.82 (0.35, 1.93) |
| **Births by women with a history of psychiatric disorders (n = 166,306)** | | | | | |
| Overall PPD | Unexposed to RA | 11300 | 292.74 | 1.00 (Ref.) | 1.00 (Ref.) |
|  | Exposed to RA | 54 | 273.78 | 0.66 (0.28, 1.53) | 0.65 (0.28, 1.53) |
| Postpartum depression | Unexposed to RA | 4858 | 122.01 | 1.00 (Ref.) | 1.00 (Ref.) |
|  | Exposed to RA | 24 | 117.87 | 0.55 (0.16, 1.95) | 0.55 (0.15, 1.95) |
| Other psychiatric disorders postpartum | Unexposed to RA | 5390 | 139.63 | 1.00 (Ref.) | 1.00 (Ref.) |
|  | Exposed to RA | 30 | 152.17 | 1.00 (0.33, 3.04) | 0.98 (0.32, 3.05) |

Abbreviations: HRs, hazard ratios; CI, confidence interval; PPD, postpartum psychiatric disorders; RA, rheumatoid arthritis; Ref., reference.

^a^ Adjusted for maternal age at delivery, calendar year of childbirth, parity, maternal highest education levels at delivery, and maternal marital status at delivery.

^b^ These analyses were conducted in Denmark and Sweden. We excluded women without register links to their mother (n=358,363), and who did not have a sister (n=406,852), resulting in 1,839,049 women being included.

**Table S8.** Hazard ratios and 95% confidence intervals for postpartum psychiatric disorders within 90 days after childbirth according to rheumatoid arthritis before childbirth after adjusting for potential explanatory factors

|  | Overall PPD | Postpartum depression | Other psychiatric disorders postpartum |
| --- | --- | --- | --- |
|  | HRs (95% CI) | HRs (95% CI) | HRs (95% CI) |
| **All births** | | | |
| Base model ^a^ (n = 3,486,020) | 1.39 (1.18, 1.64) | 1.35 (1.05, 1.75) | 1.67 (1.35, 2.06) |
| Base model + Preterm birth (n = 3,486,020) | 1.34 (1.14, 1.58) | 1.33 (1.03, 1.72) | 1.58 (1.28, 1.96) |
|  |  |  |  |
| Base model ^b^ (n = 3,469,425) | 1.36 (1.16, 1.61) | 1.32 (1.01, 1.71) | 1.65 (1.33, 2.05) |
| Base model +Small gestational age (n = 3,469,425) | 1.36 (1.15, 1.60) | 1.31 (1.01, 1.70) | 1.64 (1.32, 2.03) |
|  |  |  |  |
| Based model ^c^ (n = 3,516,849) | 1.39 (1.18, 1.64) | 1.37 (1.07, 1.77) | 1.66 (1.34, 2.05) |
| Based model + Stillbirth (n = 3,516,849) | 1.37 (1.16, 1.61) | 1.37 (1.06, 1.77) | 1.61 (1.30, 1.99) |
| Based model + Placental abruption (n = 3,516,849) | 1.39 (1.18, 1.64) | 1.37 (1.06, 1.77) | 1.65 (1.32, 2.04) |
| Based model + Pre-eclampsia (n = 3,516,849) | 1.38 (1.17, 1.63) | 1.36 (1.06, 1.76) | 1.64 (1.33, 2.03) |
| **Births by women without a history of psychiatric disorders** | | | |
| Base model (n = 3,194,307) | 1.51 (1.16, 1.96) | 1.58 (1.04, 2.41) | 1.60 (1.14, 2.26) |
| Base model + Preterm birth (n = 3,194,307) | 1.44 (1.11, 1.88) | 1.56 (1.03, 2.37) | 1.50 (1.07, 2.12) |
|  |  |  |  |
| Base model (n = 3,179,109) | 1.45 (1.10, 1.90) | 1.52 (0.99, 2.34) | 1.54 (1.08, 2.19) |
| Base model +Small gestational age (n = 3,179,109) | 1.43 (1.09, 1.88) | 1.52 (0.99, 2.33) | 1.52 (1.06, 2.16) |
|  |  |  |  |
| Based model (n = 3,223,478) | 1.52 (1.17, 1.98) | 1.65 (1.09, 2.48) | 1.59 (1.13, 2.24) |
| Based model + Stillbirth (n = 3,223,478) | 1.48 (1.14, 1.93) | 1.64 (1.09, 2.47) | 1.53 (1.09, 2.15) |
| Based model + Placental abruption (n = 3,223,478) | 1.52 (1.17, 1.97) | 1.64 (1.09, 2.48) | 1.58 (1.12, 2.23) |
| Based model + Pre-eclampsia (n = 3,223,478) | 1.51 (1.16, 1.96) | 1.63 (1.08, 2.46) | 1.57 (1.12, 2.21) |
| **Births by women with a history of psychiatric disorders** | | | |
| Base model (n = 291,713) | 0.89 (0.72, 1.09) | 0.84 (0.61, 1.16) | 1.15 (0.88, 1.52) |
| Base model + Preterm birth (n = 291,713) | 0.87 (0.71, 1.07) | 0.83 (0.60, 1.15) | 1.13 (0.86, 1.48) |
|  |  |  |  |
| Base model (n = 290,316) | 0.88 (0.72, 1.09) | 0.82 (0.59, 1.14) | 1.17 (0.89, 1.53) |
| Base model + Small gestational age (n = 290,316) | 0.88 (0.72, 1.09) | 0.82 (0.59, 1.14) | 1.17 (0.89, 1.53) |
|  |  |  |  |
| Based model (n = 293,371) | 0.88 (0.72, 1.09) | 0.84 (0.60, 1.15) | 1.15 (0.88, 1.51) |
| Based model + Stillbirth (n = 293,371) | 0.87 (0.71, 1.07) | 0.84 (0.60, 1.15) | 1.12 (0.85, 1.47) |
| Based model + Placental abruption (n = 293,371) | 0.88 (0.72, 1.09) | 0.84 (0.61, 1.15) | 1.15 (0.88, 1.51) |
| Based model + Pre-eclampsia (n = 293,371) | 0.88 (0.72, 1.08) | 0.83 (0.60, 1.15) | 1.15 (0.87, 1.50) |

Abbreviations: HRs, hazard ratios; CI, confidence interval; PPD, postpartum psychiatric disorders; RA, rheumatoid arthritis.

Adjusted for maternal age at delivery, calendar year of birth, parity, maternal highest education levels at delivery, and maternal marital status at childbirth for the base model.

^a^ We excluded participants with missing or unlikely short (<22 weeks in Denmark and in Sweden) or long gestational ages (>45 weeks in Denmark; >46 weeks in Sweden).

^b^ Small for gestational age was defined as less than the tenth percentile of birth weight for gestational age based on a Nordic reference curve for normal fetal growth.

^c^ Information on stillbirth, placental abruption, and pre-eclampsia was not missing, thus the comparison base model was our base model from our main analyses.

**Table S9.** Hazard ratios and 95% confidence intervals for postpartum psychiatric disorders within 90 days after childbirth according to rheumatoid arthritis before childbirth, in analyses restricted to primiparous women (n = 1,444,760)

|  | Number of events / person-years in women with RA | Adjusted HRs ^a^ (95% CI) |
| --- | --- | --- |
| **All births (n = 1,444,760)** | | |
| Overall PPD | 65/852 | 1.31 (1.03, 1.67) |
| Postpartum depression | 24/860 | 1.23 (0.82, 1.83) |
| Other psychiatric disorders postpartum | 40/852 | 1.60 (1.18, 2.19) |
| **Births by women without a history of psychiatric disorders (n = 1,322,105)** | | |
| Overall PPD | 29/732 | 1.60 (1.11, 2.30) |
| Postpartum depression | 12/736 | 1.75 (0.99, 3.08) |
| Other psychiatric disorders postpartum | 17/732 | 1.71 (1.06, 2.75) |
| **Births by women with a history of psychiatric disorders (n = 122,625)** | | |
| Overall PPD | 36/119 | 0.75 (0.54, 1.04) |
| Postpartum depression | 12/124 | 0.62 (0.35, 1.09) |
| Other psychiatric disorders postpartum | 23/119 | 1.02 (0.67, 1.53) |

Abbreviations: HRs, hazard ratios; CI, confidence interval; PPD, postpartum psychiatric disorders; RA, rheumatoid arthritis.

^a^ Adjusted for maternal age at delivery, calendar year of birth, parity, maternal highest education levels at delivery, and maternal marital status at childbirth.

**Table S10.** Hazard ratios and 95% confidence intervals for postpartum psychiatric disorders within 90 days after childbirth according to rheumatoid arthritis before childbirth in stratified analyses

|  | Number of events / person-years in women with RA | Adjusted HRs ^a^ (95% CI) | *P*-value for the  interaction term |
| --- | --- | --- | --- |
| **All births ^b^** | | | |
| Diabetes | | | |
| No | 133/1,928 | 1.36 (1.14, 1.61) | 0.22 |
| Yes | 13/97 | 1.91 (1.11, 3.30) |  |
| Pre-eclampsia | | | |
| No | 135/1,929 | 1.38 (1.17, 1.64) | 0.95 |
| Yes | 11/97 | 1.36 (0.75, 2.45) |  |
| **Births by women without a history of psychiatric disorders** | | | |
| Diabetes |  |  |  |
| No | 49/1,673 | 1.41 (1.06, 1.87) | 0.02 |
| Yes | 7/82 | 3.25 (1.54, 6.84) |  |
| Pre-eclampsia | |  |  |
| No | 51/1,673 | 1.49 (1.13, 1.96) | 0.72 |
| Yes | 5/81 | 1.74 (0.72, 4.20) |  |
| **Births by women with a history of psychiatric disorders** | | | |
| Diabetes |  |  |  |
| No | 84/256 | 0.89 (0.72, 1.10) | 0.78 |
| Yes | 6/16 | 0.85 (0.38, 1.89) |  |
| Pre-eclampsia | |  |  |
| No | 84/256 | 0.89 (0.72, 1.10) | 0.78 |
| Yes | 6/16 | 0.77 (0.34, 1.71) |  |

Abbreviations: HRs, hazard ratios; CI, confidence interval; RA, rheumatoid arthritis; PPD, postpartum psychiatric disorders.

^a^ Adjusted for maternal age at delivery, calendar year of childbirth, parity, maternal highest education levels at delivery, and maternal marital status at delivery.

^b^ Considering the limited number of cases, we only conducted the analyses for the overall postpartum psychiatric disorders.

**Table S11.** Baseline characteristics of the propensity score matching sub-cohort (N = 16,814)

|  | Births of women | | | | |  |
| --- | --- | --- | --- | --- | --- | --- |
|  | With RA  (N = 8,407) | |  | Without RA  (N = 8,407) | | *P-*value |
|  | No. | % |  | No. | % |  |
| **Maternal characteristics** | | | | | |  |
| Study country | | | | | |  |
| Denmark | 4,297 | 51.11 |  | 4,297 | 51.11 | 1.00 |
| Finland | 215 | 2.56 |  | 215 | 2.56 |  |
| Sweden | 3,895 | 46.33 |  | 3,895 | 46.33 |  |
| Maternal age at delivery (years) | | | | | |  |
| <25 | 735 | 8.74 |  | 735 | 8.74 | 1.00 |
| 25-29 | 2,271 | 27.02 |  | 2,282 | 27.01 |  |
| 30-34 | 3,163 | 37.62 |  | 3,158 | 37.56 |  |
| ≥35 | 2,238 | 26.62 |  | 2,232 | 26.55 |  |
| Maternal country of origin ^a^ | | | | | |  |
| Denmark/Sweden | 4,262 | 52.03 |  | 4,458 | 55.64 | <0.0001 |
| Other countries | 3,929 | 47.96 |  | 3,626 | 44.26 |  |
| Unknown | <5 | 0.01 |  | 8 | 0.10 |  |
| Maternal BMI in early pregnancy (kg/m^2^) ^a^ | | | | | |  |
| <18.5 | 204 | 2.49 |  | 173 | 2.11 | <0.01 |
| 18.5-24.9 | 3,728 | 45.51 |  | 3,821 | 46.64 |  |
| 25.0-29.9 | 1,654 | 20.19 |  | 1,576 | 19.24 |  |
| ≥30.0 | 876 | 10.69 |  | 777 | 9.48 |  |
| Unknown | 1,730 | 21.12 |  | 1845 | 22.52 |  |
| Marital status at delivery | | | | | |  |
| Single, widowed, or divorced | 4,710 | 56.03 |  | 4,408 | 52.43 | <0.0001 |
| Married or in a registered partnership | 3,690 | 43.89 |  | 3,955 | 47.04 |  |
| Unknown | 7 | 0.08 |  | 44 | 0.52 |  |
| Maternal highest education at delivery | | | | | |  |
| Primary and lower secondary | 1,226 | 14.58 |  | 1,114 | 13.25 | <0.0001 |
| Upper secondary | 3,449 | 41.03 |  | 3,290 | 39.13 |  |
| University | 3,668 | 43.63 |  | 3,831 | 45.57 |  |
| Unknown | 64 | 0.76 |  | 172 | 2.05 |  |
| Maternal smoking in early pregnancy | | | | | |  |
| No | 7,122 | 84.72 |  | 7,185 | 85.46 | 0.34 |
| Yes | 957 | 11.38 |  | 898 | 10.68 |  |
| Unknown | 328 | 3.90 |  | 324 | 3.85 |  |
| Parity | | | | | |  |
| Primiparous | 3,533 | 42.02 |  | 3,532 | 42.01 | 1.00 |
| Multiparous | 4,874 | 57.98 |  | 4,875 | 57.99 |  |
| Psychiatric disorders before childbirth | | | | | |  |
| No | 7,221 | 85.89 |  | 7,631 | 90.77 | <0.0001 |
| Yes | 1,186 | 14.11 |  | 776 | 9.23 |  |
| Family history of psychiatric disorders ^a^ | | | | |  |  |
| No | 6,631 | 80.94 |  | 6,087 | 74.30 | <0.0001 |
| Yes | 1,040 | 12.70 |  | 836 | 10.21 |  |
| Unknown ^b^ | 521 | 6.36 |  | 1,269 | 15.49 |  |
| Diabetes | | | | | |  |
| No | 7,984 | 94.97 |  | 8,171 | 97.19 | <0.0001 |
| Yes | 423 | 5.03 |  | 236 | 2.81 |  |
| Pre-eclampsia | | | | | |  |
| No | 7,996 | 95.11 |  | 8,124 | 96.63 | <0.0001 |
| Yes | 411 | 4.89 |  | 283 | 3.37 |  |
| Placental abruption | | | | | |  |
| No | 8,363 | 99.48 |  | 8,384 | 99.73 | 0.01 |
| Yes | 44 | 0.52 |  | 23 | 0.27 |  |
| **Characteristics of the birth** | | | | | |  |
| Calendar year of birth | | | | | |  |
| 1995-2000 | 759 | 9.03 |  | 751 | 8.93 | 1.00 |
| 2001-2005 | 2,077 | 24.70 |  | 2,084 | 24.79 |  |
| 2006-2010 | 3,017 | 35.89 |  | 3,019 | 35.91 |  |
| 2011-2015 | 2,554 | 30.38 |  | 2,553 | 30.37 |  |
| Small for gestational age | | | | | | |
| No | 7,280 | 86.60 |  | 7,440 | 88.50 | <0.0001 |
| Yes | 1,009 | 12.00 |  | 831 | 9.88 |  |
| Unknown | 118 | 1.40 |  | 136 | 1.62 |  |
| Preterm birth (gestational<37 weeks) | | | | | | |
| No | 7,675 | 91.30 |  | 7,907 | 94.05 | <0.0001 |
| Yes | 667 | 7.93 |  | 407 | 4.84 |  |
| Unknown ^c^ | 65 | 0.77 |  | 93 | 1.11 |  |
| Stillbirth |  |  |  |  |  |  |
| No | 8,367 | 99.52 |  | 8,380 | 99..68 | 0.11 |
| Yes | 40 | 0.48 |  | 27 | 0.32 |  |

Abbreviations: RA, rheumatoid arthritis; No., number; BMI, body-mass index.

^a^ Available only in Sweden and Denmark.

^b^ There was no register link to the biological mother or father.

^c^ Includes the missing or unlikely short (<22 weeks in Denmark and in Sweden), or long gestational ages (>45 weeks in Denmark; >46 weeks in Sweden).

**Table S12.** Hazard ratios and 95% confidence intervals for postpartum psychiatric disorders within 90 days after childbirth according to rheumatoid arthritis before childbirth in the propensity-matched sub-cohort

| Outcome | Exposure | No. | % | Incidence rate  (per 1000 person-years) | Crude model |  | | Adjusted model ^a^ |
| --- | --- | --- | --- | --- | --- | --- | --- | --- |
|  |  |  |  |  | HRs (95% CI) |  |  | HRs (95% CI) |
| **All births** | | | | | | | | |
| Overall PPD | Unexposed to RA | 107 | 1.3 | 52.2 | 1.00 (Ref.)  1.00 | |  | 1.00 (Ref.)  1.00 |
|  | Exposed to RA | 150 | 1.8 | 73.5 | 1.46 (1.05, 2.04) | |  | 1.34 (1.04, 1.71) |
| Postpartum depression | Unexposed to RA | 37 | 0.4 | 17.9 | 1.00 (Ref.)  1.00 | |  | 1.00 (Ref.)  1.00 |
|  | Exposed to RA | 63 | 0.8 | 30.6 | 1.71 (1.14, 2.56) | |  | 1.57 (1.04, 2.36) |
| Other psychiatric disorders postpartum | Unexposed to RA | 60 | 0.7 | 28.8 | 1.00 (Ref.)  1.00 | |  | 1.00 (Ref.)  1.00 |
|  | Exposed to RA | 87 | 1.0 | 42.1 | 1.46 (1.05, 2.04) | |  | 1.42 (1.02, 1.99) |
| **Births by women without a history of psychiatric disorders** | | | | | | | | |
| Overall PPD | Unexposed to RA | 54 | 0.7 | 28.9 | 1.00 (Ref.)  1.00 | |  | 1.00 (Ref.)  1.00 |
|  | Exposed to RA | 57 | 0.8 | 32.2 | 1.12 (0.77, 1.62) | |  | 1.10 (0.75, 1.60) |
| Postpartum depression | Unexposed to RA | 16 | 0.2 | 8.5 | 1.00 (Ref.) | |  | 1.00 (Ref.) |
|  | Exposed to RA | 23 | 0.3 | 13.0 | 1.52 (0.80, 2.88) | |  | 1.47 (0.78, 2.78) |
| Other psychiatric disorders postpartum | Unexposed to RA | 35 | 0.5 | 18.7 | 1.00 (Ref.) | |  | 1.00 (Ref.) |
|  | Exposed to RA | 34 | 0.5 | 19.2 | 1.03 (0.64, 1.65) | |  | 1.02 (0.63, 1.64) |
| **Births by women with a history of psychiatric disorders** | | | | | | | | |
| Overall PPD | Unexposed to RA | 53 | 6.8 | 293.0 | 1.00 (Ref.) | |  | 1.00 (Ref.) |
|  | Exposed to RA | 93 | 7.8 | 339.6 | 1.15 (0.82, 1.62) | |  | 1.12 (0.80, 1.57) |
| Postpartum depression | Unexposed to RA | 21 | 2.7 | 112.1 | 1.00 (Ref.) | |  | 1.00 (Ref.) |
|  | Exposed to RA | 40 | 3.4 | 140.5 | 1.25 (0.74, 2.12) | |  | 1.17 (0.68, 2.00) |
| Other psychiatric disorders postpartum | Unexposed to RA | 24 | 3.1 | 132.7 | 1.00 (Ref.) | |  | 1.00 (Ref.) |
|  | Exposed to RA | 53 | 4.5 | 190.0 | 1.42 (0.88, 2.31) | |  | 1.42 (0.88, 2.31) |

Abbreviations: HRs, hazard ratios; CI, confidence interval; No. number; PPD, postpartum psychiatric disorders; RA, rheumatoid arthritis; Ref., reference.

^a^ Adjusted for maternal highest education levels at delivery and maternal marital status at delivery.

**Table S13.** Hazard ratios and 95% confidence intervals for the associations between rheumatoid arthritis before childbirth and postpartum psychiatric disorders within 90 days after childbirth among women with a personal history of psychiatric disorders, further stratified by previous use of psychiatric medication

| Outcome | Exposure | No. | Incidence rate  (per 1000 person-years) | Crude model | Adjusted model ^a^ | |
| --- | --- | --- | --- | --- | --- | --- |
|  |  |  |  | HRs (95% CI) | HRs (95% CI) |  |
| **Women without previous use of psychiatric medication (n = 62,385, 32.3%)** | | | | | |  |
| Overall PPD | Unexposed to RA | 3,578 | 243.8 | 1.00 (Ref.) | 1.00 (Ref.) |  |
|  | Exposed to RA | 8 | 264.9 | 1.08 (0.54, 2.16) | 1.06 (0.51, 2.23) |  |
| Postpartum depression | Unexposed to RA | 1,312 | 86.8 | 1.00 (Ref.) | 1.00 (Ref.) |  |
|  | Exposed to RA | 2 | 63.7 | 0.73 (0.18, 2.94) | 0.46 (0.06, 3.27) |  |
| Other psychiatric disorders postpartum | Unexposed to RA | 1,739 | 118.5 | 1.00 (Ref.) | 1.00 (Ref.) |  |
|  | Exposed to RA | 6 | 198.7 | 1.67 (0.75, 3.71) | 1.68 (0.75, 3.75) |  |
| **Women with previous use of psychiatric medication ^b^ (n = 192,632, 67.7%)** | | | | | |  |
| Overall PPD | Unexposed to RA | 17,370 | 602.2 | 1.00 (Ref.) | 1.00 (Ref.) |  |
|  | Exposed to RA | 72 | 613 | 1.02 (0.81,1.28) | 0.91 (0.72, 1.15) |  |
| Postpartum depression | Unexposed to RA | 7,569 | 246.8 | 1.00 (Ref.) | 1.00 (Ref.) |  |
|  | Exposed to RA | 31 | 245.9 | 0.99 (0.70, 1.42) | 0.85 (0.59, 1.21) |  |
| Other psychiatric disorders postpartum | Unexposed to RA | 7,729 | 267.9 | 1.00 (Ref.) | 1.00 (Ref.) |  |
|  | Exposed to RA | 40 | 340.5 | 1.27 (0.93, 1.73) | 1.20 (0.88, 1.64) |  |

Abbreviations: HRs, hazard ratios; CI, confidence interval; No. number; PPD, postpartum psychiatric disorders; RA, rheumatoid arthritis; Ref., reference.

^a^ Adjusted for maternal age at delivery, calendar year of childbirth, parity, maternal highest education levels at delivery, and maternal marital status at delivery.

^b^ We used the information on the treatment of psychiatric disorders only available in Sweden during 2005-2014 and in Finland during 1995-2013.


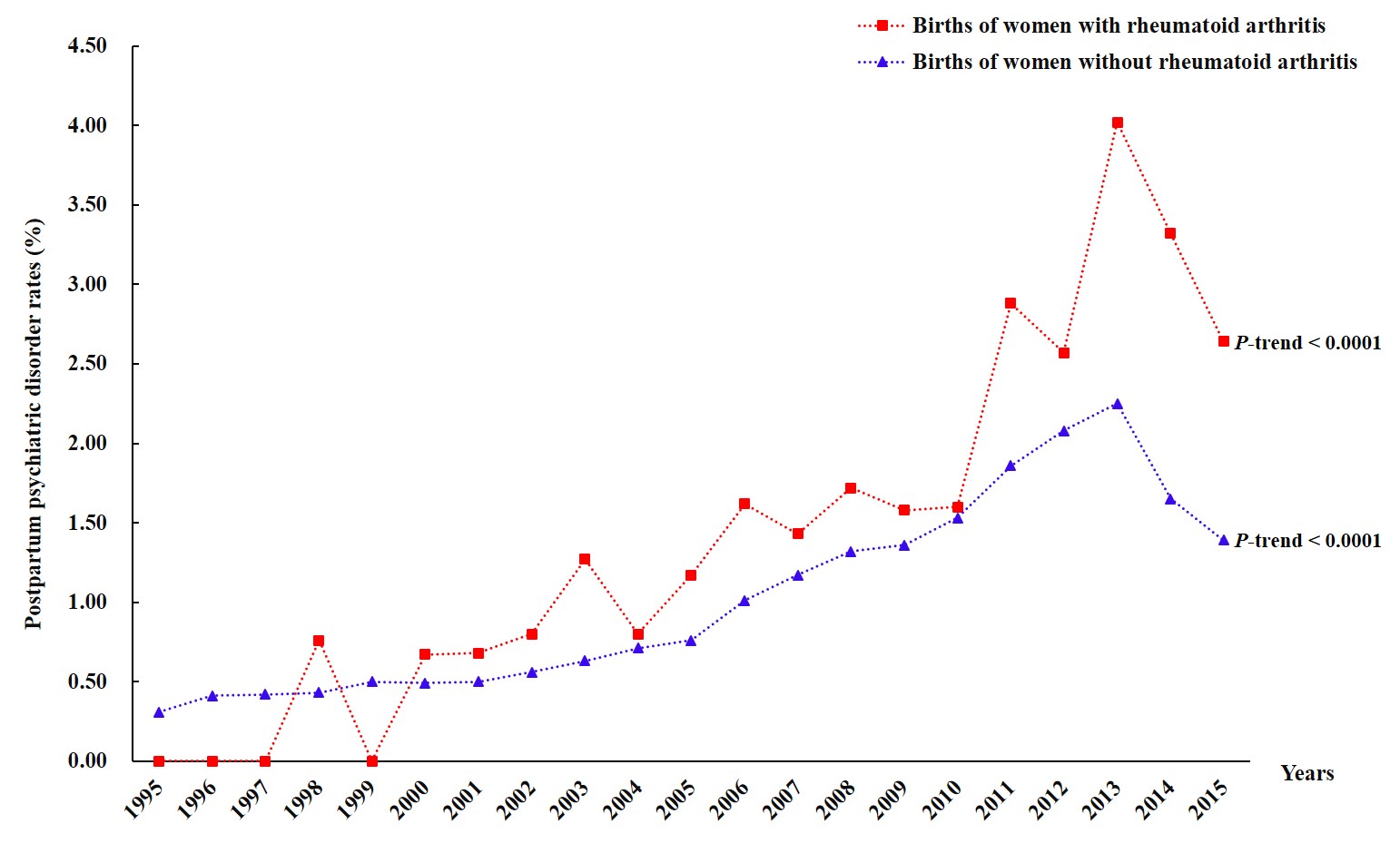
**Figure S1.** The time trend of the postpartum psychiatric disorder rates over the study period in the exposed and the unexposed group

**Figure S2.** Hazard ratios and 95% confidence intervals for postpartum psychiatric disorders within 365 days after childbirth according to clinical rheumatoid arthritis (rheumatoid arthritis diagnosed before childbirth) and preclinical rheumatoid arthritis (rheumatoid arthritis diagnosed after childbirth)


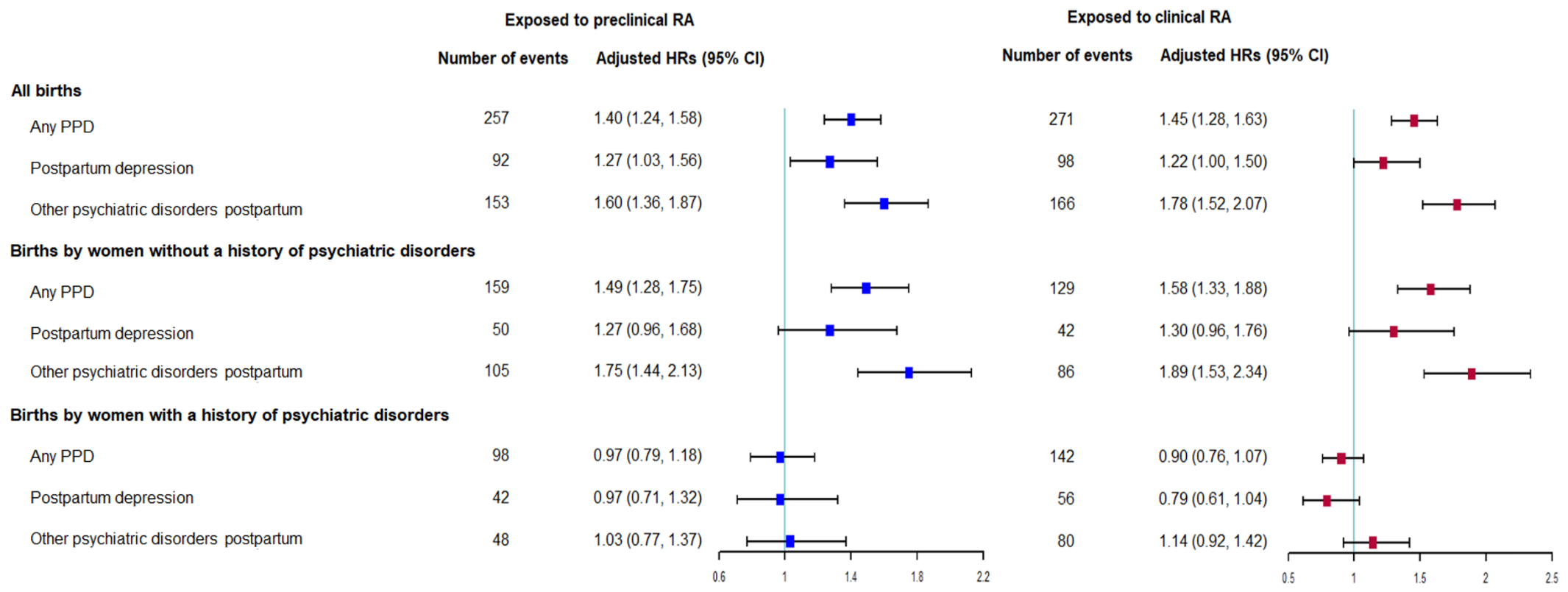


Abbreviations: HRs, hazard ratios; CI, confidence interval; PPD, postpartum psychiatric disorders; RA, rheumatoid arthritis.

Adjusted for maternal age at delivery, calendar year of childbirth, parity, maternal highest education levels at delivery, and maternal marital status at delivery.

The reference group consists of births by women who unexposed to clinical RA and preclinical RA.

**Figure S3.** Hazard ratios and 95% confidence intervals for postpartum psychiatric disorders within 365 days after childbirth according to clinical rheumatoid arthritis (rheumatoid arthritis diagnosed before childbirth) and preclinical rheumatoid arthritis (rheumatoid arthritis diagnosed in the first year after childbirth)


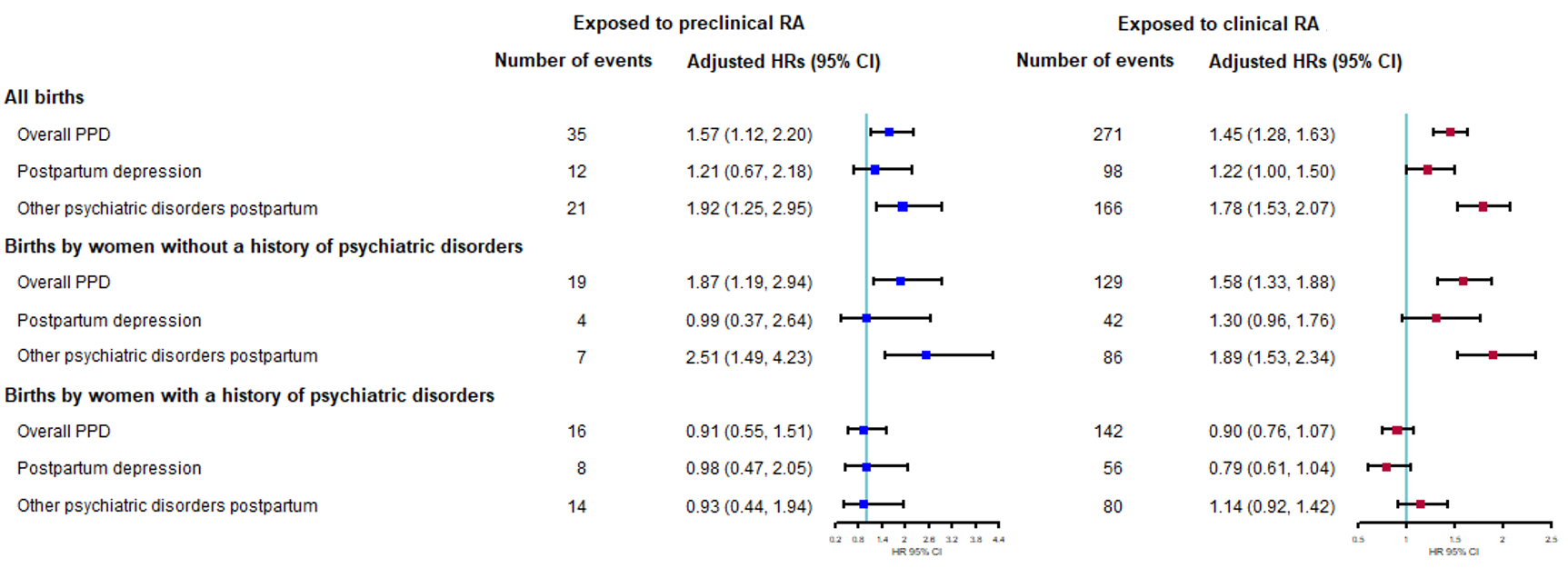


Abbreviations: HRs, hazard ratios; CI, confidence interval; PPD, postpartum psychiatric disorders; RA, rheumatoid arthritis.

Adjusted for maternal age at delivery, calendar year of childbirth, parity, maternal highest education levels at delivery, and maternal marital status at delivery.

The reference group consists of births by women who were unexposed to both clinical RA and preclinical RA.

**Figure S4.** Hazard ratios and 95% confidence intervals for the associations between rheumatoid arthritis before childbirth and postpartum psychiatric disorders within the first 365 days after delivery, stratified by a personal history of psychiatric disorders


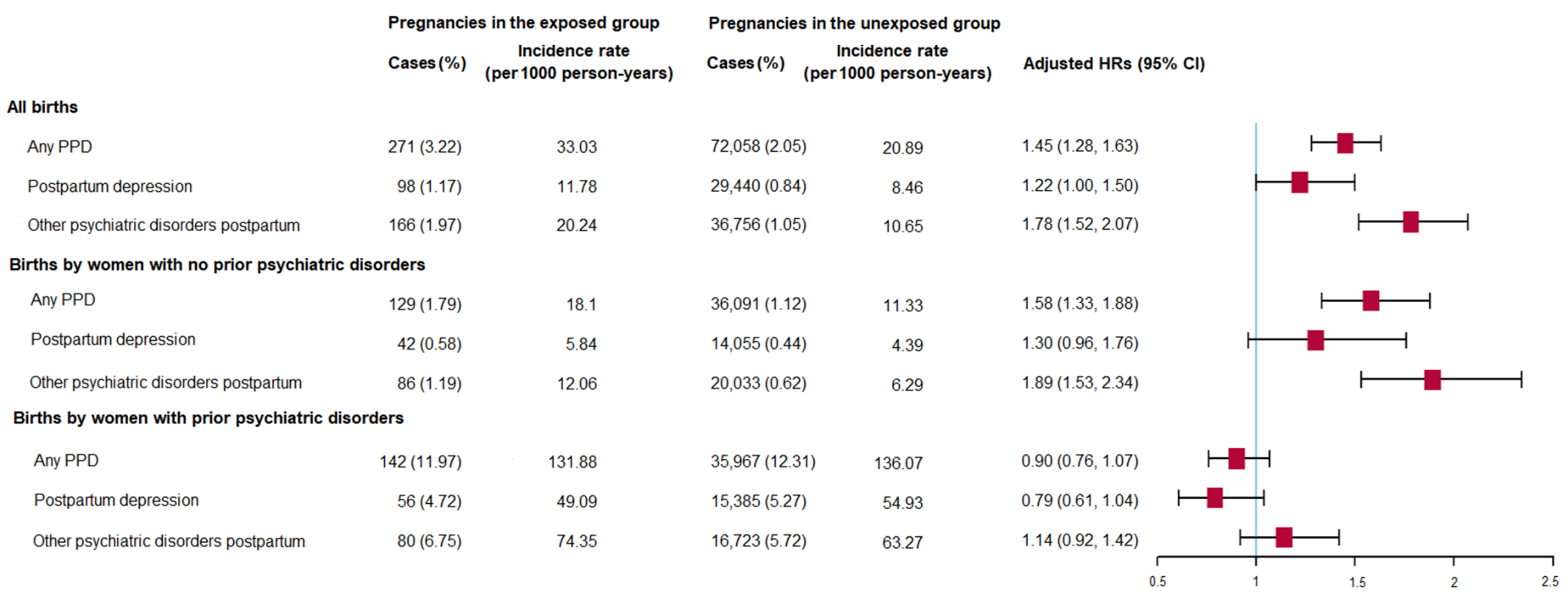


Note: Abbreviations: HRs, hazard ratios; CI, confidence interval; PPD, postpartum psychiatric disorders; RA, rheumatoid arthritis.

^a^ Adjusted for maternal age at delivery, calendar year of childbirth, parity, maternal highest education levels at delivery, and maternal marital status at delivery.
